# Predicting postoperative recurrence in colorectal cancer using MRI-derived radiomics features and their correlation with the tumor immune microenvironment

**DOI:** 10.1093/gastro/goag039

**Published:** 2026-05-18

**Authors:** Jie Zhao, Ming Li, Miao Sun, Yanjie Xin, Kai Zhao, Juxian Li, Xue Qiao, Zonghui Liang, Lei Yang, Huijie Jiang

**Affiliations:** Department of Radiology, The Second Affiliated Hospital of Harbin Medical University, Harbin, Heilongjiang, 150086, P. R. China; Department of Clinical Laboratory, Shandong Provincial Hospital affiliated to Shandong First Medical University, Jinan, Shandong, 210000, P. R. China; Department of Radiology, The Second Affiliated Hospital of Harbin Medical University, Harbin, Heilongjiang, 150086, P. R. China; Department of Radiology, The Second Affiliated Hospital of Harbin Medical University, Harbin, Heilongjiang, 150086, P. R. China; Department of Radiology, The Second Affiliated Hospital of Harbin Medical University, Harbin, Heilongjiang, 150086, P. R. China; Department of Radiology, The Second Affiliated Hospital of Harbin Medical University, Harbin, Heilongjiang, 150086, P. R. China; Department of Radiology, The Second Affiliated Hospital of Harbin Medical University, Harbin, Heilongjiang, 150086, P. R. China; Department of Radiology, Shanghai Jing’an District Central Hospital, Shanghai, 200000, P. R. China; Department of Orthopedics, The First Affiliated Hospital of Harbin Medical University, Harbin, Heilongjiang, 150086, P. R. China; State Key Laboratory of Frigid Zone Cardiovascular Diseases (SKLFZCD), Harbin Medical University, Harbin, Heilongjiang, 150086, P. R. China; Department of Radiology, The Second Affiliated Hospital of Harbin Medical University, Harbin, Heilongjiang, 150086, P. R. China

**Keywords:** colorectal cancer, postoperative recurrence, radiomics, MRI, tumor immune microenvironment

## Abstract

**Background:**

The recurrence of colorectal cancer (CRC) after surgery poses a significant threat to patient recovery, but current prediction methods lack sensitivity. In this study, we aimed to develop a radiomics-based model for recurrence prediction and to investigate its association with the tumor immune microenvironment.

**Methods:**

A retrospectively cohort study was conducted with 197 patients from the Second Affiliated Hospital of Harbin Medical University (Harbin, China; training set) and 130 patients from Shandong First Medical University Affiliated Central Hospital (Jinan, China; validation set). Clinical characteristics and blood biomarkers (alpha-fetoprotein, carcinoembryonic antigen, and cancer antigen 19-9) were compared between the recurrence and nonrecurrence groups, and magnetic resonance imaging (MRI)-derived radiomics features were extracted by using 3D Slicer and PyRadiomics. After this, LASSO regression was used to identify key features, and univariate analysis was used to retain statistically significant predictors. A multivariate logistic regression model was then developed to predict recurrence, and the relationship between radiomics features and immune cells was subsequently explored by using a CRC mouse model.

**Results:**

There were no significant differences in the clinical characteristics between recurrence and nonrecurrence groups. The initial model, comprising 10 features, achieved an area under the curve (AUC) of 0.99 in the training set but only 0.6 in the validation set. After excluding six features, the refined model, which included original gray level co-occurrence matrix sum entropy (OGSE), log-sigma-3-0-mm-3D neighborhood gray tone difference matrix Busyness (LNB), original first order total energy (OFTE), wavelet-LLH gray level size zone matrix zone variance (WGZV), achieved an AUC of 0.98 in the validation set, outperforming traditional blood biomarkers. In a murine model, regulatory T cells exhibited a strong positive correlation with OGSE (*r *= 0.84, *P *< 0.001) and moderate correlations with OFTE (*r *= 0.41, *P *= 0.035) and WGZV (*r *= 0.52, *P *= 0.007), indicating a link between radiomics features and immune cell infiltration.

**Conclusions:**

MRI-derived radiomics features, particularly OGSE, LNB, OFTE, and WGZV, can effectively predict postoperative CRC recurrence. These features are correlated with the tumor immune microenvironment, supporting the use of radiomics as a noninvasive tool for tumor assessment in CRC.

## Introduction

Colorectal cancer (CRC) is one of the most common malignancies worldwide and a leading cause of cancer-related mortality. Although surgical resection remains the cornerstone of CRC treatment, postoperative recurrence continues to be a major threat to patient survival and quality of life. Studies have demonstrated that postoperative recurrence is influenced not only by the intrinsic properties of tumor cells but also by the dynamics of the tumor microenvironment (TME), particularly its immune components [1]. The TME is composed of tumor and immune cells, fibroblasts, vasculature, and the extracellular matrix, and plays a pivotal role in tumor progression, metastasis, and therapeutic responses [2].

Dynamic changes in the TME immune landscape are closely linked to immune evasion, resistance to therapy, and recurrence after surgery. In particular, the imbalance among tumor-associated macrophages (TAMs), regulatory T cells (Tregs), and cytotoxic T lymphocytes (CTLs) plays a crucial role in immune escape and tumor recurrence. TAMs promote immune tolerance due to their immunosuppressive properties, and the accumulation of Tregs exacerbates this immune suppression, thereby increasing the risk of postoperative recurrence [3]. Furthermore, the dysfunction or absence of CTLs impedes the immune system’s ability to eradicate residual tumor cells. Although the role of the immune microenvironment in postoperative recurrence is increasingly being recognized, there remains a lack of effective, noninvasive methods to monitor immune changes, especially for the early detection of recurrence. The current reliance on serum biomarkers such as carcinoembryonic antigen (CEA), carbohydrate antigen 19-9 (CA19-9), and alpha-fetoprotein (AFP) is limited by insufficient sensitivity and specificity, rendering them inadequate for reliable clinical prediction [4–6].

Radiomics, a novel imaging analysis technique, has exhibited great potential for noninvasive diagnosis of tumors, prognosis prediction, and treatment monitoring [7]. By extracting large-scale, high-dimensional features from medical images, radiomics quantifies the morphological, textural, and functional characteristics of tumors, which are closely associated with the biological behavior of tumors. Recently, radiomics has gained significant research attention, particularly in predicting the postoperative CRC recurrence [8, 9]. However, despite its increasing application in tumor prediction and monitoring, the relationship between radiomics features and the immune microenvironment remains largely underexplored.

In this study, we aimed to explore a radiomics-based approach integrated with immunological insights in order to predict postoperative CRC recurrence. We initially collected magnetic resonance imaging (MRI) T2-weighted imaging data from clinical CRC cohorts and performed radiomics analysis to identify features associated with postoperative recurrence. A predictive model for recurrence within two years post-surgery was then constructed based on these features and compared with conventional serum biomarkers (AFP, CA199, and CEA). Additionally, an orthotopic murine CRC model was established to validate the biological relevance of these radiomics features. Immune cell populations, such as TAMs, Tregs, and CTLs, were analyzed within the TME to explore the underlying immune mechanisms. By correlating radiomics features with immune cell dynamics, we sought to provide new insights into the immunological mechanisms that drive postoperative CRC recurrence.

**Figure 1:**
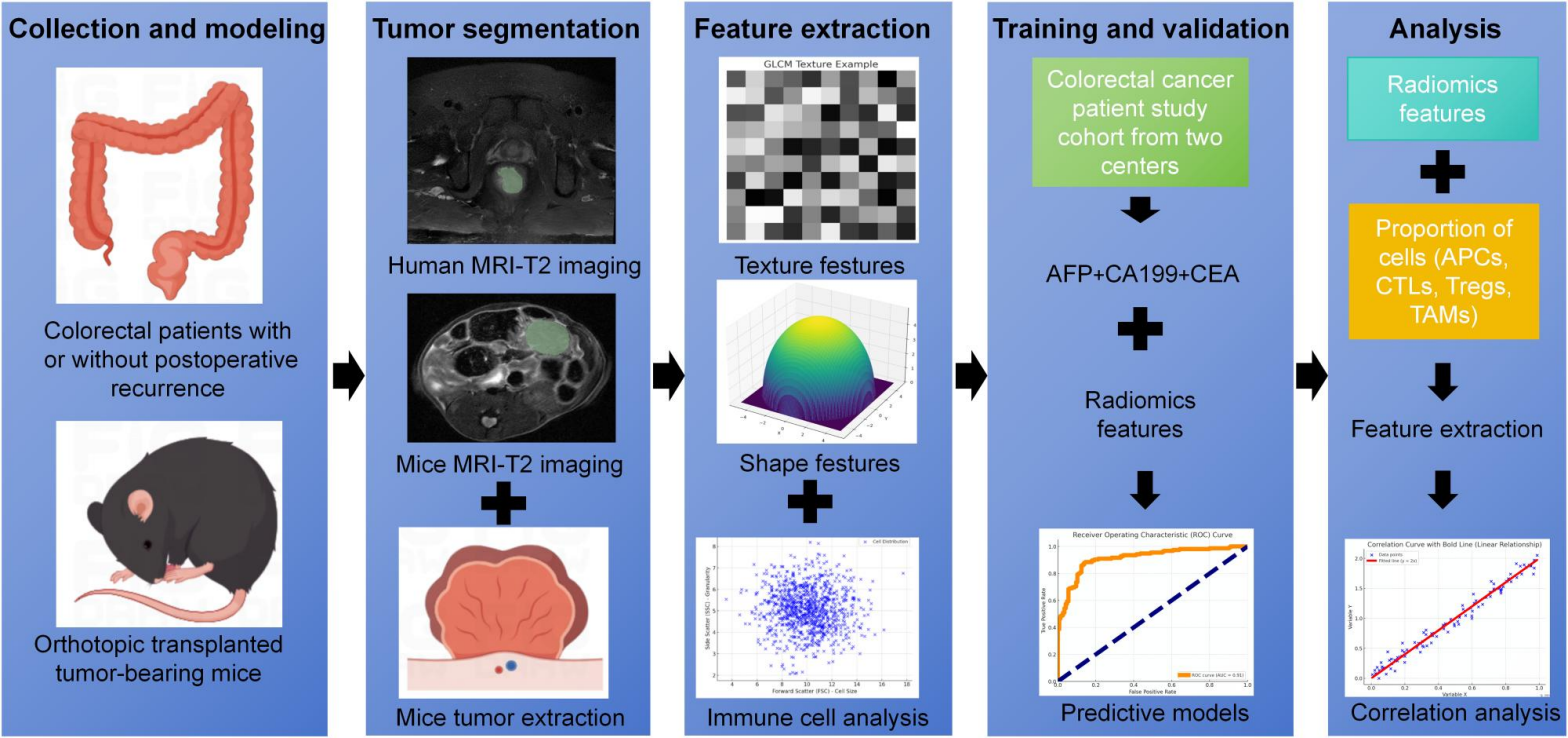
Study workflow and methodology. The schematic illustrates the comprehensive workflow of the study and details, the steps from data collection to model development and biological validation. CRC patient cohorts with or without postoperative recurrence are included for clinical imaging analysis. MRI T2-weighted images from both human and murine models are used for tumor segmentation and feature extraction, encompassing texture and shape features, as well as immune cell analysis. Radiomics features are extracted from the imaging data and used to build predictive models, which are validated by using serum biomarkers (AFP, CA199, and CEA). The biological relevance of these radiomics features is further explored in a murine orthotopic CRC model, with histological analysis (TUNEL staining for apoptotic cells and flow cytometry for immune cells) conducted to assess the tumor microenvironment. The correlation between radiomics features and immune cell populations is analyzed to explore the underlying biological mechanisms associated with postoperative recurrence in CRC. CRC, colorectal cancer; AFP, Alpha-fetoprotein; CA19-9, Carbohydrate Antigen 19-9; CEA, carcinoembryonic antigen; MRI, magnetic resonance imaging; GLCM, gray-level co-occurrence matrix; APCs, antigen-presenting cells; CTLs, cytotoxic T lymphocytes; Tregs, regulatory T cells; TAMs, tumor-associated macrophages.

Although the role of the immune microenvironment in CRC recurrence has received increasing attention, the deployment of effective and noninvasive methods for monitoring and predicting postoperative recurrence still presents a significant challenge. By integrating radiomics with immunology, this study developed an innovative, noninvasive framework (Fig. 1) for the early prediction of CRC recurrence and real-time assessment of the immune microenvironment. This framework comprises clinical‑imaging data acquisition, radiomics feature extraction, predictive modeling, and biological validation. It may provide new strategies for personalized treatment and advance precision medicine in CRC management.

## Materials and methods

### Study design and cohorts

Clinical and pathological data were retrospectively collected from patients with CRC who underwent curative-intent surgical resection between January 2017 and December 2021. The study was designed as a retrospective cohort analysis, and all included patients were identified from existing medical records at two independent medical centers. Preoperative imaging and clinical data were systematically extracted from historical archives, and postoperative outcomes were assessed through a review of follow-up records.

The training cohort included patients from the Second Affiliated Hospital of Harbin Medical University (Harbin, China), whereas the validation cohort included patients from the Shandong First Medical University Affiliated Central Hospital (Jinan, China). The patients were invited to participate in the study once they were diagnosed with primary CRC and scheduled for curative-intent surgical resection at both centers. All participants underwent standardized preoperative MRI scanning and comprehensive clinical evaluation, including laboratory testing and tumor marker assessment, as part of routine clinical care and prospective data collection.

The inclusion criteria were as follows: (i) age 18 years or older; (ii) histopathologically confirmed primary CRC; (iii) no prior anti-tumor treatment (including chemotherapy, radiotherapy, or surgery); and (iv) scheduled preoperative MRI as part of standard clinical care, ensuring feasibility of radiomics feature extraction, and commitment to planned follow-up for postoperative recurrence monitoring. The exclusion criteria were (i) presence of distant metastases at baseline; (ii) prior or concurrent malignancies; or (iii) withdrawal of consent or failure to complete imaging or follow-up visits for reasons unrelated to the study protocol. Notably, the requirement for complete imaging and clinical data was integrated into the study protocol at the time of patient enrollment, rather than being applied retrospectively.

This study was conducted in accordance with the ethical standards established by the institutional review boards of both participating hospitals: ethical approval was granted by the Clinical Ethics Committee of Shandong First Medical University Affiliated Central Hospital (approval number: 2021-012-13) and the Clinical Ethics Committee of the Second Affiliated Hospital of Harbin Medical University (approval number: YJSKY2021-322).

### Clinical data collection

Clinical data were prospectively collected from patients with CRC who underwent surgical resection at the Second Affiliated Hospital of Harbin Medical University (training set) and the Shandong First Medical University Affiliated Central Hospital (validation set) as gathered from medical records, preoperative assessments, and imaging studies. Basic demographic information, including age, sex, and medical history, was collected from all patients. Additionally, clinical parameters such as preoperative laboratory values (including platelet count and hemoglobin levels) were recorded, and blood samples were obtained for analysis of tumor markers (including AFP, CEA, and CA199), which were measured by using standard clinical assays.

Preoperative MRI scans were acquired for all patients by using a standardized imaging protocol. The scans included T2-weighted imaging sequences that were used for tumor segmentation and subsequent radiomics feature extraction. Radiologists reviewed the imaging data for tumor location, size, and other relevant characteristics, and radiomics features were extracted from the tumor region of interest (ROI) during segmentation.

Clinicopathological characteristics were extracted from pathology reports and surgical records, including TNM staging based on the AJCC 8th edition, resection margin status (R0: negative; R1: positive), and histological subtype. Surgical procedures were classified as either laparoscopic or open resection. Additionally, postoperative adjuvant therapy, including chemotherapy and/or radiotherapy, was documented and included in statistical analysis.

All patients were prospectively followed up with for up to 24 months after surgery, with follow-up scheduled every 3 months. Surveillance included a physical examination, serum marker testing, and imaging (computed tomography or MRI). The primary endpoint was tumor recurrence within 2 years, as confirmed by radiological and/or histopathological evidence. These clinicopathological and treatment-related variables were later included in a multivariate logistic regression model to assess their effect on recurrence risk and determine the independent predictive value of the selected radiomics features.

### Preclinical model

To validate the biological relevance of radiomics features in the tumor immune microenvironment, we established a murine orthotopic model with CRC. Thirty-five female C57BL/6 mice (8 weeks old) were subcutaneously implanted with MC38 CRC cells obtained from the American Type Culture Collection. The cells were cultured in RPMI-1640 medium supplemented with 10% fetal bovine serum and 1% penicillin/streptomycin and maintained at 37 °C in a 5% CO_2_ incubator. Cells were passaged during the logarithmic growth phase by using 0.25% trypsin/EDTA solution for detachment. For cryopreservation, the cells were suspended in freezing medium containing 10% dimethyl sulfoxide and stored at −80°C.

The mice were housed in a controlled environment at 25 °C with a 12-h light/dark cycle and provided *ad libitum* access to drinking water and standard rodent chow. Each mouse was monitored weekly for signs of distress or illness. Tumor modeling was initiated by subcutaneously injecting 1 × 10^6^ MC38 cells into the flank of each mouse, and tumor growth was monitored at regular intervals by comparing tumor volume. When tumors reached 200–300 mm^3^, an MRI was performed to assess their characteristics.

### MRI acquisition

Human imaging data were obtained by using a 3 T Siemens Skyra MRI system employing axial T2-weighted turbo spin-echo sequences (TR/TE = 3,200/85 ms, slice thickness = 3 mm, and matrix size = 384 × 384). The tumor ROIs were manually delineated by two radiologists with over 5 years of experience each using 3D Slicer (version 5.2.2), and interobserver agreement was quantified by using the Dice similarity coefficient. For the murine model, MRI was performed on a 9.4 T Bruker BioSpec MRI system, with sagittal T2-weighted scans (TR/TE = 2,500/50 ms, and resolution = 0.1 × 0.1 × 0.5 mm³). High-resolution MRI is crucial for detecting small tumor foci and monitoring tumor growth, particularly in murine liver metastases.

### Radiomics analysis

Radiomics feature extraction was performed by using PyRadiomics software (version 3.0.1), which generated 1,304 features, including shape, texture, and wavelet features. Prior to the analysis, all features were *Z*-score-normalized to eliminate differences in scale. LASSO regression was then applied for feature selection, and 10-fold cross-validation was used to identify the optimal regularization parameter (λ = 0.032). This procedure reduces overfitting while selecting the most informative features associated with postoperative recurrence prediction. Features with a *P*-value < 0.05 from logistic regression were selected for multivariate modeling. Model performance was evaluated by using receiver operating characteristic (ROC) curves, calculating the area under the curve (AUC), sensitivity, and specificity, and applying DeLong’s test to compare the radiomics model with traditional serum biomarkers (CEA, AFP, and CA199).

### TUNEL staining

To assess apoptosis, TUNEL staining was performed on 5-µm tumor tissue sections. An *In Situ* Cell Death Detection Kit (Roche, Beijing, China) was used to identify the apoptotic cells, after which the proportion of apoptotic cells in different tumor tissues was compared.

### Flow cytometry

For immune cell profiling, tumor tissues were processed into single-cell suspensions and stained with the following antibodies: anti-TREM2 (1/60, ab245227, Abcam) for TREM2^+^ macrophages, anti-CD68 (1/50, ab283654, Abcam) for general macrophage identification, anti-CD8 (1/50, ab217344, Abcam) and anti-CD3 (1/70, ab16669, Abcam) for CTLs, and anti-CD25 (1/70, ab264557, Abcam) and anti-CD4 (1/70, ab207755, Abcam) for Tregs. Flow cytometric data were acquired by using a BD FACSymphony A3 flow cytometer and analyzed by using FlowJo software (version 10.8).

### Statistical analysis

All statistical analyses were conducted using R software (version 4.2.2). Continuous variables were compared between groups by using either the Mann–Whitney *U*-test or the *t*-test, depending on the data distribution. Chi-squared or Fisher’s exact test was used to assess categorical variables. Univariate analysis was performed to evaluate the relationship between the individual radiomics features and postoperative recurrence, where differences were considered statistically significant at *P < *0.05. For continuous variables, logistic regression models were constructed to examine the association between the features and recurrence. Significant features from the univariate analysis were incorporated into multivariate models for further validation.

Multivariate logistic regression was used for this model construction, where we employed a stepwise regression method to select the most relevant features based on statistical significance. The final model was assessed using Akaike’s Information Criterion (AIC) to evaluate goodness-of-fit, with lower AIC values indicating a better-fitting model. The model performance was validated in the training cohort and evaluated by using an independent validation cohort, and the sensitivity, specificity, and positive predictive value were also calculated. ROC curves were plotted, and the AUC was calculated to evaluate the discriminatory ability of the model. After this, DeLong’s test was used to compare the performance of the radiomics model with that of the traditional biomarkers (CEA, AFP, and CA199).

To evaluate whether radiomics features provide independent prognostic value beyond conventional clinical factors, we constructed multivariate logistic regression models that incorporated both radiomics features and clinicopathological covariates. Specifically, the following variables were included as covariates based on their known relevance to recurrence: tumor TNM stage, resection margin status (R0 vs R1), surgical approach (laparoscopic vs open), and adjuvant therapy (yes or no). To account for multiple comparisons, the Benjamini–Hochberg false discovery rate (FDR) correction was applied with a 5% FDR threshold for all statistical tests. Pearson’s correlation coefficient (r) was used to evaluate the relationships between radiomics features and immune cell markers, and a *P-*value < 0.05 was considered statistically significant.

## Results

### Comparison of postoperative recurrence risk between patients with different clinical characteristics

We compared the clinical characteristics of patients in the recurrence and nonrecurrence groups across training and validation sets (Table 1). The training set included 75 patients in the recurrence group and 122 patients in the nonrecurrence group, whereas the validation set consisted of 41 patients with recurrence and 89 patients without recurrence. There were no significant differences in age or gender between the recurrence and nonrecurrence groups. The mean age did not differ significantly between the recurrence and non-recurrence groups in both the training and validation sets (both *P *> 0.05; Table 1). The gender distribution was also similar, with insignificant differences.

In the training set, mean value of platelet count was slightly higher in the recurrence group than in the nonrecurrence group, whereas in the validation set, mean value of platelet count was lower in the recurrence group than in the nonrecurrence group, although these differences were not statistically significant. Hemoglobin levels were similar between the groups in both sets as well, with no statistical differences.

For the blood biomarkers AFP, CEA, and CA199, no significant differences were found between the recurrence and nonrecurrence groups in either the training or validation sets. The mean AFP, CEA, and CA199 levels did not differ significantly between the two groups (*P > *0.05) either. The resection margins and surgical approaches were significantly different between the recurrence and nonrecurrence groups in the training set. The recurrence group exhibited a higher proportion of R1 resection margins (16.0% vs 4.1%, *P = *0.009) and a higher proportion of open surgeries (40.0% vs 24.6%, *P = *0.034). However, in the validation set, only differences in the resection margin and surgical approach were close to statistical significance (*P = *0.077 and *P = *0.08, respectively).

### Feature selection and analysis

**Figure 2:**
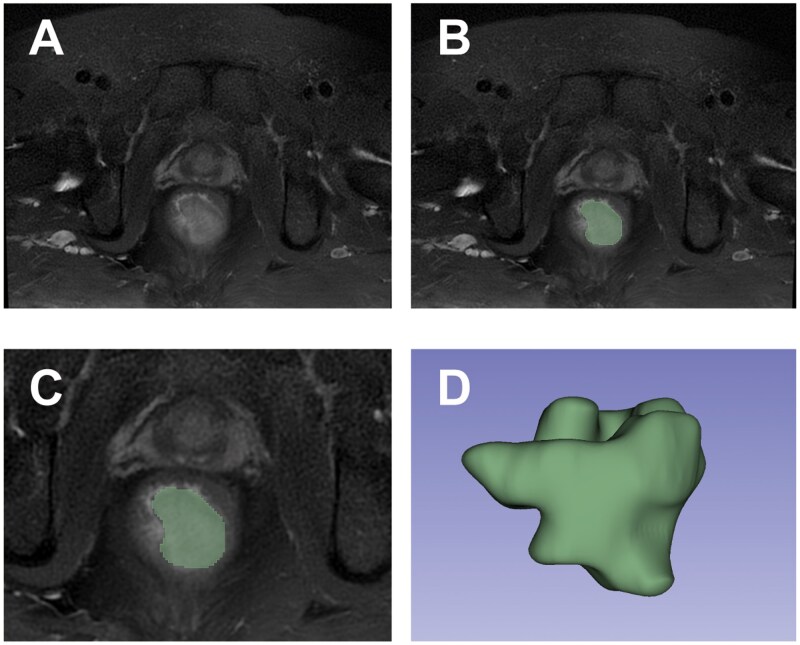
Tumor segmentation and 3D reconstruction using 3D-slicer software. (A) Axial T2-weighted MRI image of a colorectal tumor, showing the tumor without any annotations. (B) Delineation of the ROI on a single MRI slice, with the tumor’s boundary marked for radiomics feature extraction. (C) Magnified view of the segmented ROI from panel B, highlighting the tumor’s precise boundary. (D) 3D reconstruction of the tumor volume based on multi-slice ROI annotations, offering a visual representation of the tumor’s spatial morphology. Tumor segmentation and 3D reconstruction were performed using the 3D-Slicer software (www.slicer.org) to facilitate the extraction of radiomics features from the MRI data. MRI, magnetic resonance imaging; ROI, region of interest.

**Figure 3:**
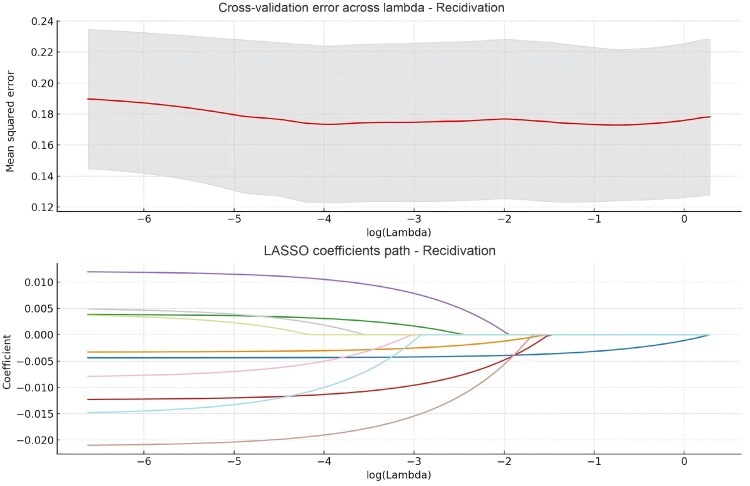
LASSO model for radiomics feature selection in recidivism prediction. Up: Cross-validation error across Lambda. The plot shows the cross-validation mean-squared error for different values of log-transformed regularization parameters (Lambda) in the LASSO model, illustrating the trend in error as Lambda increases. The red line represents the optimal model, with the shaded region indicating the standard deviation across cross-validation folds. Down: LASSO Coefficients Path. This plot demonstrates the path of the coefficients for each feature selected by the LASSO model as the regularization parameter (Lambda) changes. As Lambda increases, some coefficients shrink toward zero, indicating the selection of only the most relevant features. The different colors represent different radiomics features that were included in the model.

The selection of radiomics features for predicting postoperative recurrence in CRC followed a multi-step process, beginning with tumor segmentation and annotation, followed by statistical analysis. Initial tumor segmentation was performed on the MRI images to define the ROI (Fig. 2). This segmentation allowed the extraction of radiomics features, such as texture, shape, and statistical characteristics, which were then analyzed to identify potential predictors of postoperative recurrence. LASSO regression was then employed to refine the feature set (Fig. 3). This approach reduces dimensionality by shrinking the coefficients of less important features to zero, ultimately identifying a subset of features with nonzero coefficients. Ten key radiomics features were selected by using LASSO; these features included original shape matrix elongation (OSE), original first order interquartile range (OFIR), original gray level co-occurrence matrix sum entropy (OGSE), original firstorder mean absolute deviation (OFMAD), original gray level dependence matrix gray level nonuniformity (OGGLNU), log-sigma-3-0-mm-3D neighborhood gray tone difference matrix busyness (LNB), original firstorder robust mean absolute deviation (OFRMAD), original firstorder total energy (OFTE), original firstorder uniformity (OFU), wavelet-LLH gray level size zone matrix zone variance (WGZV).

Following LASSO feature selection, univariate analysis was conducted to assess the relationship between these features and the risk of recurrence. As summarized in Table 2, statistically significant associations (*P < *0.05) were observed for most features, except for OFRMAD, indicating their potential as predictive markers for postoperative recurrence. These selected radiomics features were compared between the recurrence and nonrecurrence groups in the training and validation sets (Table 3). The training set revealed significant differences for all features. However, in the validation set, several features, including OSE, OFIR, OFMAD, OGGLNU, OFRMAD, and OFU, did not exhibit statistically significant differences between the recurrence and nonrecurrence groups. This suggests that the predictive power of these features may vary across datasets. Thus, feature selection using LASSO, univariate analysis, and intergroup comparisons identified four key radiomics features (OGSE, LNB, OFTE, and WGZV) that demonstrated significant associations with postoperative recurrence risk.

**Table 1:** Clinical characteristics comparison of postoperative recurrence risk in colorectal cancer: training and validation sets

Variable	Training set			Validation set		
	Recurrence group (*N *= 75)	Non-recurrence group (*N *= 122)	*P* **-value**	Recurrence group (*N *= 41)	Non-recurrence group (*N *= 89)	*P* **-value**
Age (years)	63.1 ± 7.2	62.7 ± 7.7	0.786	62.9 ± 6.9	62.5 ± 7.9	0.858
Gender (male %)	59.1%	49.7%	0.352	51.5%	44.4%	0.492
Plts	244.80 ± 79.42	239.68 ± 58.00	0.638	225.59 ± 65.21	254.47 ± 82.93	0.075
Hb	134.07 ± 17.18	131.95 ± 17.74	0.484	125.95 ± 17.49	129.17 ± 14.25	0.378
RBC	4.39 ± 0.61	4.43 ± 0.53	0.702	4.16 ± 0.53	4.23 ± 0.47	0.513
WBC	6.14 ± 1.33	6.19 ± 2.03	0.883	6.06 ± 1.64	6.11 ± 1.22	0.667
Lymph (%)	30.18 ± 9.25	31.72 ± 8.25	0.287	29.31 ± 8.46	31.21 ± 8.56	0.324
Abs lymph	1.84 ± 0.63	1.88 ± 0.54	0.653	1.71 ± 0.54	1.87 ± 0.54	0.184
AFP	2.92 ± 1.46	3.15 ± 1.55	0.379	5.60 ± 2.89	3.16 ± 1.55	<0.001
CEA	76.64 ± 286.48	22.01 ± 136.97	0.079	26.91 ± 77.51	12.91 ± 20.49	0.082
CA199	102.59 ± 296.32	71.11 ± 323.96	0.564	68.67 ± 215.36	63.55 ± 210.59	0.017
Tumor location, *n* (%)			0.363			0.232
Colon	41 (54.7%)	76 (62.3%)		21 (51.2%)	57 (64.0%)	
Rectum	34 (45.3%)	46 (37.7%)		20 (48.8%)	32 (36.0%)	
TNM stage n (%)			0.033			0.047
Stage I	3 (4.0%)	18 (14.8%)		4 (9.8%)	16 (18.0%)	
Stage II	18 (24.0%)	42 (34.4%)		9 (22.0%)	33 (37.1%)	
Stage III	33 (44.0%)	48 (39.3%)		18 (43.9%)	32 (36.0%)	
Stage IV	21 (28.0%)	14 (11.5%)		10 (24.4%)	8 (9.0%)	
Resection margin n (%)			0.009			0.077
R0	63 (84.0%)	117 (95.9%)		34 (82.9%)	84 (94.4%)	
R1	12 (16.0%)	5 (4.1%)		7 (17.1%)	5 (5.6%)	
Surgical approach n (%)			0.034			0.08
Laparoscopic	45 (60.0%)	92 (75.4%)		25 (61.0%)	69 (77.5%)	
Open	30 (40.0%)	30 (24.6%)		16 (39.0%)	20 (22.5%)	
Adjuvant therapy n (%)			0.014			0.069
Yes	54 (72.0%)	65 (53.3%)		28 (68.3%)	44 (49.4%)	
No	21 (28.0%)	57 (46.7%)		13 (31.7%)	45 (50.6%)	
Histologic subtype n (%)			0.426			0.801
Adenocarcinoma	59 (78.7%)	103 (84.4%)		32 (78.0%)	74 (83.1%)	
Mucinous	11 (14.7%)	12 (9.8%)		5 (12.2%)	8 (9.0%)	
Signet-ring	5 (6.7%)	7 (5.7%)		4 (9.8%)	7 (7.9%)	

Independent *t*-test or Mann–Whitney *U*-test (for continuous variables not following a normal distribution) was used to compare differences between the Recurrence and non-Recurrence groups; chi-squared test was used for categorical variables (Gender). Plts, platelets (×10^9^/L); Hb, hemoglobin (g/L); RBC, red blood cells (×10^12^/L); WBC, white blood cells (×10^9^/L); Lym%, Lymphocyte % (%); Abs Lym, absolute lymphocyte count (×10^9^/L); AFP, alpha-fetoprotein (ng/mL); CEA, carcinoembryonic antigen (ng/mL); CA199, cancer antigen 19-9 (U/mL).

**Table 2:** Univariate analysis of radiomics features associated with postoperative recurrence in colorectal cancer patients

Radiomics feature	OR (95% confidence interval)	*P*-value
OSE	0.001 (0.000, 0.001)	0.046
OFIR	0.001 (0.000, 0.001)	0.015
OGSE	0.582 (0.025, 1.139)	0.041
OFMAD	0.002 (0.000, 0.003)	0.01
OGGLNU	0.003 (0.001, 0.006)	0.007
LNB	0.001 (0.000, 0.001)	0.046
OFRMAD	0.001 (0.000, 0.001)	0.079
OFTE	0.004 (0.001, 0.008)	0.01
OFU	0.001 (0.000, 0.001)	0.034
WGZV	0.000 (0.000, 0.000)	0.009

Univariate logistic regression was used to assess the association of each radiomics feature with postoperative recurrence in colorectal cancer patients. OR, odds ratio; OSE, elongation (Original Shape Matrix); OFIR, interquartile range (Original Firstorder); OGSE, sum entropy (Original GLCM); OFMAD, mean absolute deviation (Original Firstorder); OGGLNU, Gray level non uniformity (Original GLDM); LNB, Busyness (Log-sigma-3-0-mm-3D, NGDTM); OFRMAD, robust mean absolute deviation (Original Firstorder); OFTE, total energy (Original Firstorder); OFU, uniformity (Original Firstorder); WGZV, zone variance (Wavelet-LLH, GLSZM).

**Table 3:** Comparison of radiomics features between recurrence and non-recurrence groups in training and validation sets

Radiomics feature	Training set			Validation set		
	Recurrence group (*N *= 75)	Non-recurrence group (*N *= 122)	*P*-value	Recurrence group (*N *= 41) (mean ± SD)	Non-recurrence group (*N *= 89) (mean ± SD)	*P*-value
OSE	1,173.87 ± 533.38	998.33 ± 457.79	0.032	689.34 ± 154.06	664.09 ± 118.99	0.539
OFIR	1,656.58 ± 815.31	1,359.74 ± 615.78	0.009	1,094.84 ± 180.67	1,032.87 ± 134.83	0.092
OGSE	5.46 ± 0.74	5.24 ± 0.59	0.039	8.49 ± 1.68	7.12 ± 0.93	<0.001
OFMAD	442.76 ± 297.69	342.95 ± 179.99	0.006	211.59 ± 106.49	202.64 ± 89.81	0.889
OGGLNU	284.92 ± 184.94	218.77 ± 112.27	0.004	192.95 ± 60.68	170.59 ± 39.77	0.061
LNB	1,173.87 ± 533.38	998.33 ± 457.79	0.032	941.21 ± 30.45	917.81 ± 13.98	<0.001
OFRMAD	1,127.74 ± 533.31	970.10 ± 470.31	0.059	473.46 ± 202.63	510.17 ± 204.95	0.425
OFTE	186.97 ± 125.73	145.09 ± 76.37	0.007	84.57 ± 103.39	47.52 ± 51.22	0.018
OFU	1,236.69 ± 573.07	1,040.18 ± 476.09	0.022	743.74 ± 289.46	676.22 ± 267.16	0.286
WGZV	194,337.61 ± 276,541.05	102,297.78 ± 125,537.45	0.002	59,586.93 ± 14,849.54	55,767.70 ± 21,626.21	0.025

Note: Independent *t*-test or Mann–Whitney *U*-test (for continuous variables not following a normal distribution) was used to compare differences between the recurrence and non-recurrence groups; SD, standard deviation; OSE, elongation (Original Shape Matrix); OFIR, interquartile range (Original Firstorder); OGSE, sum entropy (Original GLCM); OFMAD, mean absolute deviation (Original Firstorder); OGGLNU, gray level non uniformity (Original GLDM); LNB, Busyness (Log-sigma-3-0-mm-3D, NGDTM); OFRMAD, robust mean absolute deviation (Original Firstorder); OFTE, total energy (Original Firstorder); OFU, uniformity (Original Firstorder); WGZV, zone variance (Wavelet-LLH, GLSZM).

### The impact of feature reduction on prediction performance

**Figure 4:**
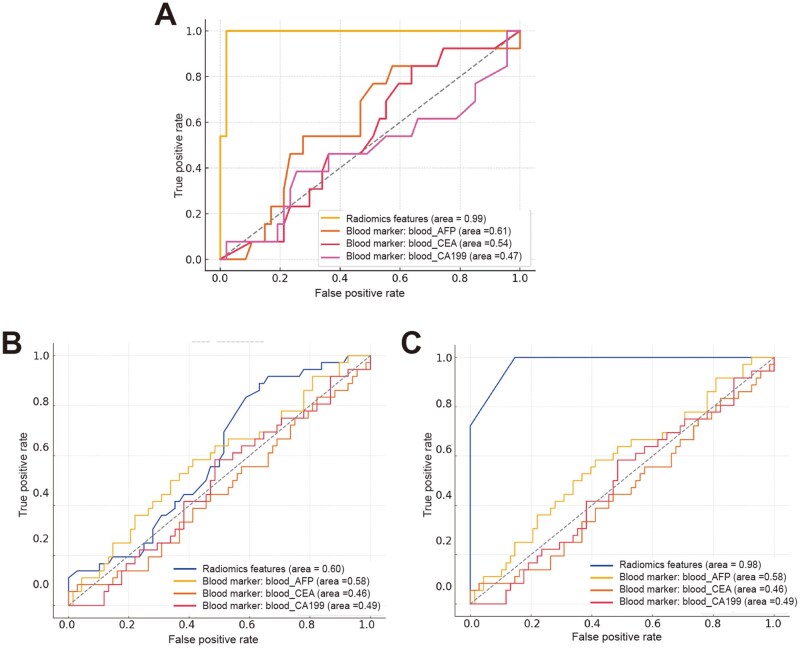
ROC curve analysis of combined radiomics features and blood biomarkers for predicting postoperative recurrence. (A) Training set ROC curves. ROC curves for combined radiomics features (orange curve) and blood biomarkers (AFP, CEA, CA199) in the training set. (B) Validation set ROC curves. ROC curves for the same predictors in the validation set. (C) Validation set ROC curves after feature reduction. After excluding six insignificant radiomics features, the four significant features (OGSE, LNB, and OFTE) were combined. The combined radiomics features (blue curve, AUC = 0.98) showed improved prediction accuracy, and blood biomarkers remained lower (AFP: AUC = 0.58, CEA: AUC = 0.46, CA199: AUC = 0.49).

We initially assessed a predictive model using all the selected radiomics features and blood biomarkers. In the training set, the combined radiomics features (AUC = 0.99) significantly outperformed the individual blood biomarkers (AFP: AUC = 0.61; CEA: AUC = 0.54; CA199: AUC = 0.47) (Fig. 4A), and in the validation set, the combined radiomics features (AUC = 0.60) provided better performance than the blood biomarkers (AFP: AUC = 0.58; CEA: AUC = 0.46; CA199: AUC = 0.49) (Fig. 4B). We then refined the model by excluding six radiomics features that exhibited no significant association with postoperative recurrence in the validation set: OSE, OFIR, OFMAD, OGGLNU, OFRMAD, and OFU. The remaining four features, OGSE, LNB, OFTE, and WGZV, were retained in the final predictive model.

As displayed in Fig. 4C, the combination of these four features yielded a significant improvement in prediction accuracy, achieving an AUC of 0.98 in the validation set. This finding suggests that OGSE, LNB, OFTE, and WGZV were closely associated with postoperative recurrence and strongly contributed to the predictive power of the model. In contrast, the blood biomarkers exhibited lower AUC values (AFP: AUC = 0.58; CEA: AUC = 0.46; CA199: AUC = 0.49), further highlighting the robustness of the radiomics model.

### Independent predictive value of radiomics features for postoperative recurrence

Univariate and multivariate logistic regression models were used to assess the association between the radiomics features and postoperative recurrence (Table 4). In the training set, univariate analysis revealed that all four features (OGSE, LNB, OFTE, and WGZV) were significantly associated with recurrence. Specifically, OGSE (odds ratio [OR] = 2.87, *P *< 0.001), LNB (OR = 2.34, *P = *0.006), and OFTE (OR = 2.71, *P = *0.001) exhibited a strong predictive potential. After adjustment for clinical covariates (TNM stage, resection margin status, surgical approach, and adjuvant therapy), the multivariate model confirmed that all four features remained statistically significant independent predictors: OGSE (OR = 2.43, *P* = 0.001), OFTE (OR = 2.11, *P* = 0.008), LNB (OR = 1.97, *P* = 0.021), and WGZV (OR = 1.85, *P* = 0.032).

**Table 4:** Comparison of radiomics features between recurrence and non-recurrence groups in training sets

Variable	Unadjusted OR	Unadjusted 95% CI	Unadjusted *P*-value	Adjusted OR	Adjusted 95% CI	Adjusted *P*-value
OGSE	2.87	1.73–4.76	<0.001	2.43	1.42–4.16	0.001
LNB	2.34	1.28–4.30	0.006	1.97	1.11–3.48	0.021
OFTE	2.71	1.54–4.77	0.001	2.11	1.22–3.65	0.008
WGZV	2.02	1.12–3.65	0.025	1.85	1.05–3.26	0.032

Radiomics features included OGSE, LNB, OFTE, and WGZV. Univariate odds ratios (ORs), 95% confidence intervals (CIs), and *P*-values were obtained from separate logistic regression models without adjustment for clinical covariates. Multivariate models were adjusted for known recurrence-associated clinical factors, including tumor TNM stage (III/IV vs I/II), resection margin status (R1 vs R0), surgical approach (open vs laparoscopic), and receipt of adjuvant therapy (yes vs no).

In the validation set (Table 5), after adjusting for clinical factors, OGSE (*P *= 0.015) and OFTE (*P *= 0.035) remained statistically significant, further confirming their roles as reliable predictors of recurrence. In contrast, LNB (*P *= 0.071) and WGZV (*P *= 0.083) exhibited reduced predictive power, with *P*-values > 0.05, suggesting variability in their prognostic utility across the cohorts.

**Table 5:** Univariate and multivariate logistic regression results for MRI-derived radiomics features in the validation cohort

Variable	Unadjusted OR	Unadjusted 95% CI	Unadjusted *P*-value	Adjusted OR	Adjusted 95% CI	Adjusted *P*-value
OGSE	2.65	1.33–5.31	0.006	2.34	1.18–4.67	0.015
LNB	2.12	1.09–4.13	0.027	1.85	0.95–3.60	0.071
OFTE	2.38	1.20–4.76	0.013	2.08	1.06–4.13	0.035
WGZV	1.9	1.01–3.58	0.046	1.76	0.93–3.33	0.083

Radiomics features included OGSE, LNB, OFTE, and WGZV. Univariate odds ratios (ORs), 95% confidence intervals (CIs), and *P*-values were obtained from separate logistic regression models without adjustment for clinical covariates. Multivariate models were adjusted for known recurrence-associated clinical factors, including tumor TNM stage (III/IV vs I/II), resection margin status (R1 vs R0), surgical approach (open vs laparoscopic), and receipt of adjuvant therapy (yes vs no).

**Figure 5:**
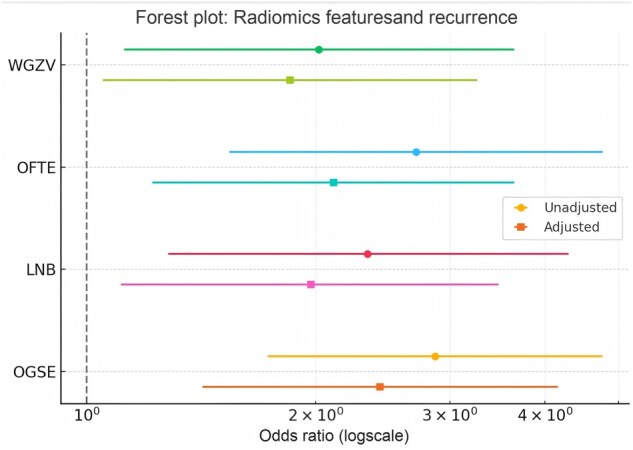
Forest plot illustrating unadjusted and adjusted ORs with 95% CIs for four MRI-derived radiomics features in predicting postoperative recurrence in colorectal cancer. Unadjusted models were based on univariate logistic regression. Adjusted models included TNM stage (III/IV vs I/II), resection margin status (R1 vs R0), surgical approach (open vs laparoscopic), and receipt of adjuvant therapy as covariates. All four radiomics features remained statistically significant independent predictors of recurrence after adjustment. CI, confidence interval; OR, odds ratio.

Figure 5 illustrates the unadjusted and adjusted ORs for the four MRI-derived radiomics features in a forest plot. Both unadjusted and adjusted models supported OGSE, LNB, OFTE, and WGZV as independent predictors of recurrence, although the adjusted models exhibited a slight reduction in the strength of the association between LNB and WGZV. Notably, OGSE and OFTE retained robust significance even after adjusting for clinical covariates, highlighting their potential clinical utility in predicting the postoperative CRC recurrence.

### Radiomics features and tumor immune microenvironment correlations

**Figure 6:**
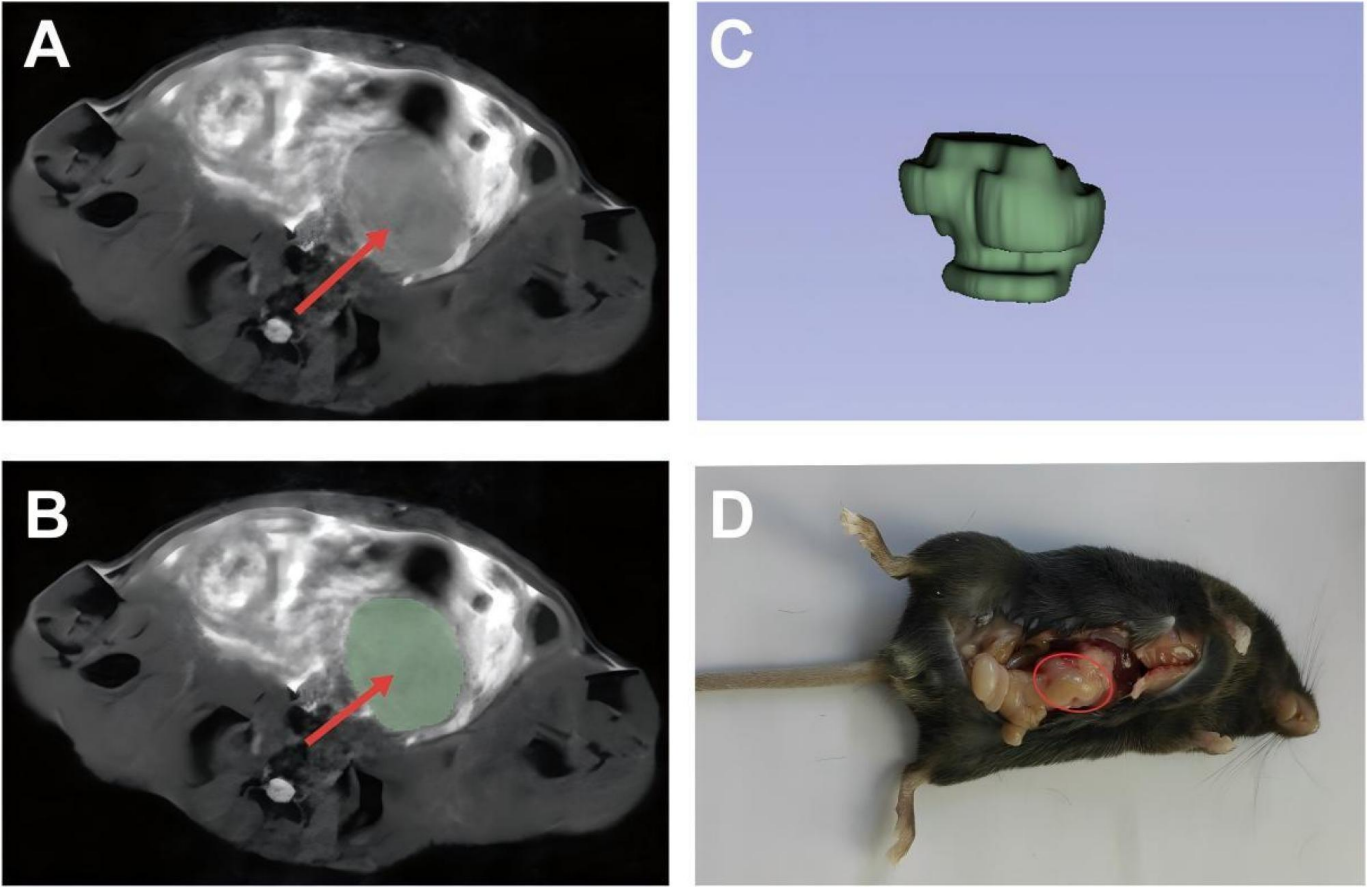
MRI target region determination in the orthotopic colorectal cancer tumor mouse model. (A) MRI image. Axial T2-weighted MRI showing the colorectal tumor in the mouse abdomen before segmentation. (B) Tumor segmentation. The tumor region is delineated (green contour) on the MRI slice for radiomics feature extraction. (C) 3D reconstruction. 3D reconstruction of the tumor volume based on MRI data, visualizing the tumor’s spatial morphology. (D) *In vivo* tumor growth. The photo shows a mouse with an implanted colorectal tumor, revealing the growing tumor mass upon dissection.

We further explored the relationship between radiomics features and immune cell populations in the TME. Using the orthotopic CRC tumor mouse model, we performed MRI segmentation (Fig. 6) and immune cell profiling using flow cytometry to assess the correlation between tumor characteristics and immune responses. Tumor segmentation was initially performed by using MRI to define the ROI (Fig. 6A and B), followed by 3D reconstruction of the tumor volume (Fig. 6C), which facilitated the extraction of radiomics features for analysis. This approach enabled a comprehensive assessment of tumor morphology in relation to immune cell infiltration.

**Figure 7:**
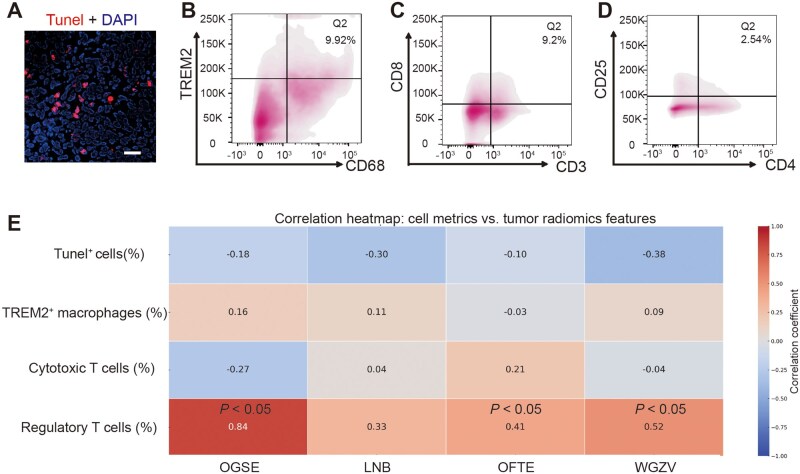
Correlation of tumor immune cell metrics with radiomics features. (A) TUNEL and DAPI staining. Representative image showing apoptotic cells (TUNEL^+^, red) in the tumor tissue, counterstained with DAPI (blue). Scale bar = 50 µm. (B) Flow cytometry of TREM2^+^ macrophages. Representative flow cytometry plot showing the percentage of TREM2^+^ macrophages (*y*-axis) vs CD68 expression (*x*-axis). (C) Flow cytometry of cytotoxic T cells. Flow cytometry plot showing the percentage of CD8^+^ cytotoxic T cells (*y*-axis) vs CD68 expression (*x*-axis). (D) Flow cytometry of regulatory T cells. Flow cytometry plot showing the percentage of CD4^+^CD25^+^ regulatory T cells (*y*-axis) vs CD3 expression (*x*-axis). (E) Correlation heatmap. Correlation coefficients between tumor immune cell metrics and radiomics features, calculated using Pearson correlation. Statistical analysis was performed with N > 30, and asterisks denote statistically significant correlations: *P < 0.05.

To characterize the immune landscape, we analyzed key immune cell populations. Tumor tissues were stained for apoptotic cells (TUNEL^+^, red), and flow cytometry was performed to evaluate the percentage of TREM2^+^ macrophages, CD8^+^ cytotoxic T cells, and CD4^+^CD25^+^ Tregs (Fig. 7A and D). This analysis allowed us to examine the correlation between tumor-associated immune cells and radiomics features. A correlation heatmap was then generated to explore the relationship between tumor immune cell metrics and radiomics features (Fig. 7E). Pearson’s correlation analysis was conducted with *N *> 30, and statistical significance was assessed. The results revealed significant correlations between specific immune cell populations and radiomics features. Notably, Tregs demonstrated strong positive correlations with OGSE (*r *= 0.84, *P *< 0.001), OFTE (*r *= 0.41, *P *= 0.035), and LNB (*r *= 0.33, *P *= 0.120), as well as a moderate positive correlation with WGZV (*r *= 0.52, *P *= 0.007). In contrast, TREM2^+^ macrophages showed a weak positive association with OGSE (*r *= 0.16, *P *= 0.085), while cytotoxic T cells exhibited a weak negative correlation with OGSE (*r *= −0.27, *P *= 0.012). These findings suggest that the TME, particularly the infiltration of Tregs, was closely linked to specific tumor imaging characteristics, underscoring the potential of radiomics to assess immune responses in tumors accurately.

## Discussion

In this study, we demonstrated that MRI-derived radiomics features, particularly OGSE, LNB, and OFTE, provided significant predictive value for postoperative recurrence in CRC, and these findings are consistent with the growing body of evidence supporting the use of radiomics as a powerful tool for noninvasive, image-based prognosis. Although the blood biomarkers AFP, CEA, and CA199 have traditionally been used for monitoring CRC recurrence, we found that they did not perform as well as the radiomics model. This highlights the potential of MRI-based radiomics to serve as a more reliable prognostic tool, especially in cases where traditional blood biomarkers fall short in detecting subtle changes in tumor behavior.

The use of MRI in predicting CRC progression has been explored in several studies over the years, with a focus on tumor size, shape, and response to therapy [10]. In previous studies, conventional MRI-based measurements, such as tumor volume and invasion depth, have been found to be correlated with clinical outcomes, including recurrence and survival rates. However, these conventional MRI techniques often lack high sensitivity that is required for detecting more subtle, underlying biological processes that contribute to tumor progression, particularly in the context of early recurrence and micro-metastasis [11].

Recent advances in radiomics have revolutionized this approach by enabling the assessment of tumor heterogeneity and complex texture patterns that reflect the underlying biology of tumors. Radiomics features can capture subtle variations in tumor tissue composition that are often missed by traditional imaging methods [12]. This approach allows for the identification of high-dimensional patterns that may be predictive of clinical outcomes, such as recurrence, long before detectable changes in tumor size occur [13–15].

One of the most promising developments in this field has been the application of texture-based features, such as OGSE [16]. These features quantify the complexity and heterogeneity of the tumor tissue, which are known to be important determinants of tumor aggressiveness and metastatic potential. Specifically, OGSE has been shown in previous studies to be associated with tumor aggressiveness, invasion, and poor prognosis in a variety of cancers, including CRC [17–19]. Our study built on this body of work, and showed that OGSE played a crucial role in predicting postoperative recurrence in CRC, which further emphasized its clinical relevance.

OGSE stood out as one of the most significant features identified in our study. As a texture feature derived from the GLCM, OGSE quantified the disorder or randomness in the pixel intensity distribution within the tumor [20]. A higher sum entropy value indicates greater complexity and heterogeneity, which have been linked to more aggressive tumor behavior [21]. In CRC, the heterogeneity of tumor tissue was associated with increased invasive potential, resistance to chemotherapy, and a higher likelihood of recurrence [22].

Previous research has demonstrated that OGSE can be a robust predictor of tumor behavior, especially in imaging modalities such as MRI, where subtle differences in tumor texture can reflect underlying changes in the TME, such as hypoxia, necrosis, and fibrosis [23]. The correlation between OGSE and Tregs in our study is consistent with these findings, as Tregs are often present in the TME and are involved in creating an immunosuppressive niche that promotes tumor progression. The positive correlation of OGSE with Tregs further reinforces the idea that radiomics features such as OGSE not only capture morphological characteristics of tumors but also provide insights into their immunological milieu [24].

One of the key strengths of this study is the integration of MRI-derived radiomics with immune cell profiling, which indeed provided a more comprehensive understanding of tumor biology. The use of LASSO regression for feature selection ensured that only the most relevant features were retained, minimizing the risk of overfitting. Moreover, the incorporation of immune cell metrics from flow cytometry allowed us to explore the complex interactions between tumor tissue and the immune microenvironment, further enhancing the predictive accuracy of our model.

The relationship between OGSE (Sum Entropy) and Tregs highlights the increasing recognition of radiomics as not just a tool for characterizing tumor morphology but also a window into the immune microenvironment. Previous studies have shown that Tregs play a pivotal role in immune suppression and the evasion of immune surveillance within the TME, particularly in CRC. For example, research by Wang *et al.* [25] has demonstrated that high levels of Tregs are associated with tumor progression, poor prognosis, and treatment resistance. The positive correlation between OGSE and Tregs in our study suggests that radiomics features, such as OGSE, can serve as indirect biomarkers of immune suppression. These findings are also consistent with the work of Huang *et al.* [26], who showed that tumor heterogeneity, as measured through radiomics, correlates with immune cell infiltration, including Tregs, in various malignancies. By reflecting Treg infiltration, OGSE may therefore offer a noninvasive tool to assess immune-related tumor characteristics, providing critical insights into tumor immunology and potential therapeutic resistance mechanisms.

In addition to the established relationship between radiomics and immune cells such as Tregs, recent advances have underscored the integration of radiomics with immune checkpoint blockade therapies. Studies by Zhang *et al.* [27] have shown that tumors with high radiomics heterogeneity often harbor an immune-excluded phenotype, where immune cells, despite being present in the TME, are unable to penetrate and mount an effective response against the tumor. This aligns with our finding that OGSE correlates with immune suppression markers, particularly Tregs. Moreover, radiomics features that capture tumor metabolic and structural heterogeneity (such as OFTE and LNB) may reflect immune evasion mechanisms. The ability of radiomics to quantify these features opens new avenues for identifying patients who might benefit from immunotherapy or combination therapies targeting both the tumor and the immune system. This concept has been further explored by Jeon *et al*. [28], who demonstrated the potential of radiomics in predicting response to immunotherapy based on tumor-immune interactions. In this context, radiomics not only enhances our understanding of tumor biology but also facilitates the development of personalized, immune-informed treatment strategies, highlighting its pivotal role in precision oncology.

Despite its strengths, this study has some limitations. First, the sample size, although reasonable, may not fully capture the heterogeneity of CRC across different stages and subtypes. Future studies should aim to validate our findings in larger, multi-center cohorts to improve the generalizability of the radiomics model. Moreover, although MRI is a powerful imaging modality, integrating radiomics features from other imaging modalities, such as CT or PET, could provide a more comprehensive understanding of tumor biology and enhance predictive accuracy. Furthermore, even though our study identified significant correlations between radiomics features and immune cells, the underlying mechanisms behind these relationships remain unclear. Future research should therefore aim to explore the mechanistic basis of these correlations, particularly the role of Tregs in influencing tumor heterogeneity and recurrence. Longitudinal studies are also needed to assess the potential of radiomics as a tool for monitoring treatment response and detecting early recurrence.

## Conclusions

This study highlights the significant predictive value of MRI-derived radiomics features, particularly OGSE, LNB, OFTE, and WGZV, in assessing postoperative recurrence risk in CRC. The correlation between radiomics features and immune cell populations, especially Tregs, suggests that MRI can provide valuable insights into the TME. As the field of radiomics continues to evolve, integrating these features with other molecular and immune profiling tools holds great promise for advancing personalized treatment strategies for CRC.
